# Retraction statement: Ubiquitin ligase SMURF1 functions as a prognostic marker and promotes growth and metastasis of clear cell renal cell carcinoma

**DOI:** 10.1002/2211-5463.13515

**Published:** 2022-11-15

The above article published online on 07 February 2017 in Wiley Online Library (https://doi.org/10.1002/2211-5463.12204), has been retracted by agreement between the Editor‐in‐Chief, John Wiley and Sons Ltd and the authors. The retraction has been agreed following an investigation based on allegations raised by a third party, which revealed that some images in this article are inappropriate duplications of images from previously published articles [1, 2, 3]. Thus, the editors consider the conclusions of this manuscript substantially compromised.
